# Supplementary material for: A classification-based generative approach to selective targeting of global slow oscillations during sleep
Source: Front Hum Neurosci. 2024 Feb 13;18:1342975. doi: 10.3389/fnhum.2024.1342975 (PMC10896842; doi:10.3389/fnhum.2024.1342975)
Supplement: Supplementary file 1 [file Data_Sheet_1.docx]

# Supplementary material

## Tables

Table S1. Sleep outcomes for our 22 participants. WASO: wake after sleep onset. For each value, time is reported in minutes.

| Total Sleep Time | $465.36\pm7.76$ |
| --- | --- |
| S1 | $22.1591\pm10.40$ |
| S2 | $199.75\pm25.92$ |
| SWS | $126.68\pm31.61$ |
| REM | $102.07\pm14.96$ |
| Sleep Onset | $14.04\pm6.71$ |
| WASO | $5.30\pm4.75$ |

Table S2. Count of SOs per participant during stage 2 and SWS.

|  | Stage 2 | | | SWS | | |
| --- | --- | --- | --- | --- | --- | --- |
| Participant ID | Total SOs | Global SOs | Non-Global SOs | Total SOs | Global SOs | Non-Global SOs |
| 1 | 12016 | 6259 | 5757 | 76568 | 35684 | 40884 |
| 2 | 9263 | 1436 | 7827 | 26372 | 2800 | 23572 |
| 3 | 11244 | 3690 | 7554 | 66459 | 17821 | 48638 |
| 4 | 15677 | 6591 | 9086 | 33090 | 4967 | 28123 |
| 5 | 16835 | 7572 | 9263 | 58246 | 16097 | 42149 |
| 6 | 12962 | 3790 | 9172 | 34982 | 7573 | 27409 |
| 7 | 5649 | 884 | 4765 | 8784 | 1468 | 7316 |
| 8 | 10035 | 4751 | 5284 | 13406 | 4136 | 9270 |
| 9 | 13702 | 4526 | 9176 | 49634 | 17210 | 32424 |
| 10 | 15203 | 7244 | 7959 | 56065 | 20443 | 35622 |
| 11 | 10689 | 2934 | 7755 | 26649 | 7098 | 19551 |
| 12 | 2086 | 0 | 2086 | 4164 | 100 | 4064 |
| 13 | 9911 | 2173 | 7738 | 20128 | 2304 | 17824 |
| 14 | 7224 | 1064 | 6160 | 16009 | 1114 | 14895 |
| 15 | 6917 | 554 | 6363 | 18798 | 2774 | 16024 |
| 16 | 10908 | 6055 | 4853 | 44873 | 17759 | 27114 |
| 17 | 14994 | 8447 | 6547 | 110766 | 58393 | 52373 |
| 18 | 16840 | 8868 | 7972 | 77381 | 32620 | 44761 |
| 19 | 8636 | 1676 | 6960 | 18880 | 3727 | 15153 |
| 20 | 15408 | 7624 | 7784 | 46514 | 16624 | 29890 |
| 21 | 17589 | 9563 | 8026 | 84597 | 42546 | 42051 |
| 22 | 7607 | 1727 | 5880 | 28711 | 8877 | 19834 |
| SUM | 251395 | 97428 | 153967 | 921076 | 322135 | 598941 |

Table S3. Count and median duration (s) of global and non-global SOs during stage 2, SWS, and combined stages.

|  | Stage 2 | | SWS | | Stage2 + SWS | |
| --- | --- | --- | --- | --- | --- | --- |
|  | Count | Median duration (s) | Count | Median duration (s) | Count | Median duration (s) |
| Global SOs | 97428 | 1.1200 | 322135 | 1.0920 | 419563 | 1.0990 |
| Non-global SOs | 153967 | 1.1480 | 598941 | 1.0210 | 752908‬ | 1.0420 |
| Total SOs | 251395 | 1.2060 | 921076 | 1.0465 | 1172471 | 1.0720 |

Table S4. Optimal symmetric electrode montage and parameters of the stimulation waveforms.

| Function | parameters | Electrode montage |
| --- | --- | --- |
| W_1_ | $A=$0.1631  $f=$0.0471  $\emptyset=$0.4966  $O=$0.3685 | F1, F2, P3, P4 |
| W_2_ | $A_{1}=$0.2290  $f_{1}=$0.1280  $\emptyset_{1}=$1.1215  $A_{2}=$0.0801  $f_{2}=$1.5466  $\emptyset_{2}=$-2.4277  $A_{3}=$0.0969  $f_{3}=$1.1933  $\emptyset_{3}=$1.4360  $O=$0.4303 | F5, F6, P1, P2 |
| W_3_ | $A=$0.0385  $f=$3.0311  $\emptyset=$1.9479  $D=$52.9285  $O=$0.2919 | F7, F8, O1, O2 |
| W_4_ | $p_{1}=0.5377$  $p_{2}=0.5707$  $p_{3}=0.6044$  $p_{4}=-0.2658$  $\emptyset=-0.6812$ | Fp1, Fp2, P5, P6 |

## Figures

**
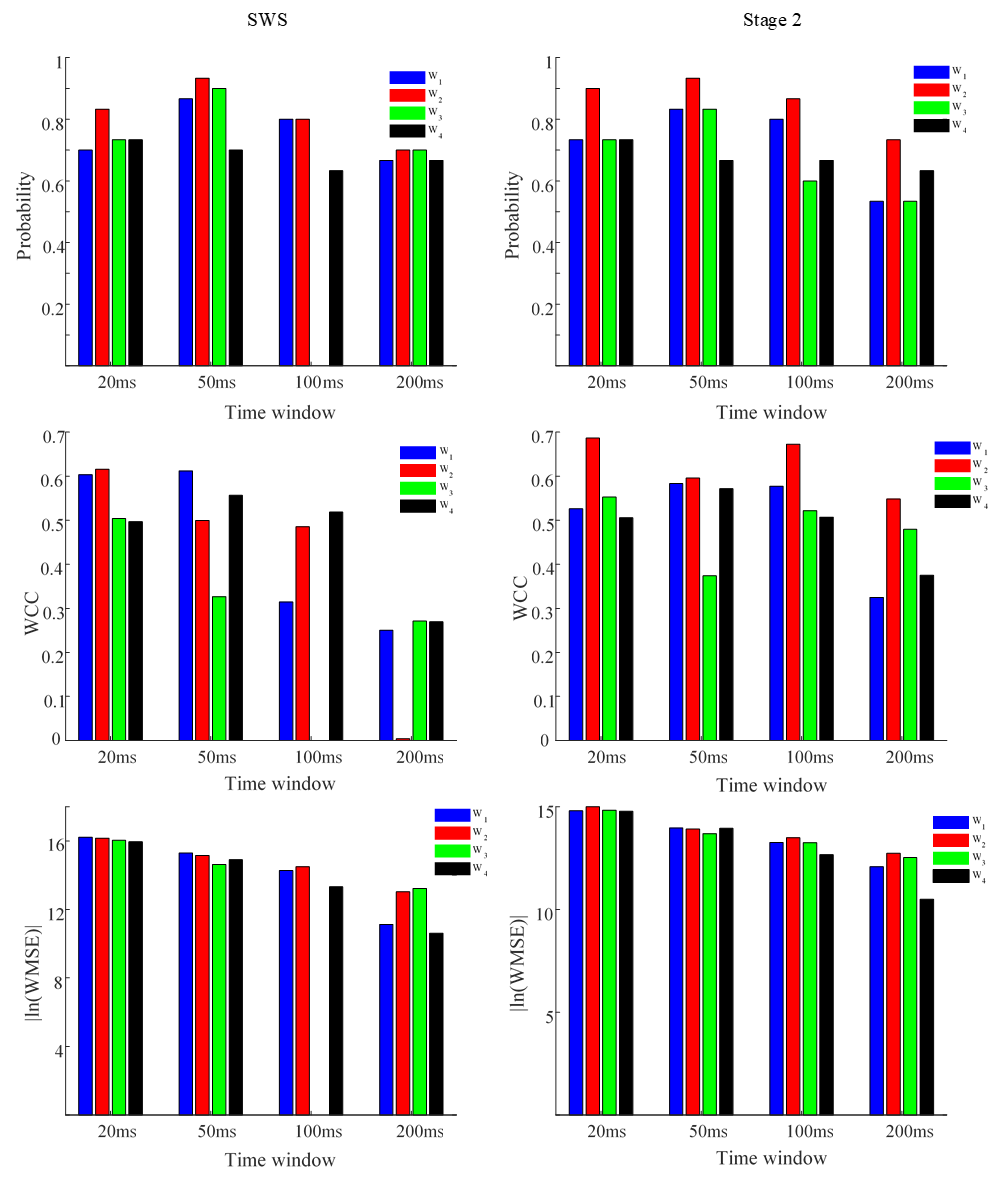
**

Figure S1. Performance metrics of stage 2 and SWS. First to third rows show probability of classification, WCC and |ln(WMSE)|. Left and right columns show results regarding to stage 2 and SWS respectively. Calculating absolute value of logarithm of WMSE is due to make the values comparable visually.
